# American Doctors

**Published:** 1871-05

**Authors:** 


AMERICAN DOCTORS.—According to the present census there are seventy four thousand Doctors in this country.

